# Supplementary material for: Increased 20-HETE Signaling Suppresses Capillary Neurovascular Coupling After Ischemic Stroke in Regions Beyond the Infarct
Source: Front Cell Neurosci. 2021 Nov 8;15:762843. doi: 10.3389/fncel.2021.762843 (PMC8606525; doi:10.3389/fncel.2021.762843)
Supplement: Supplementary file 1 [file Data_Sheet_1.docx]

Supplementary Material

# Supplementary Figures and Tables


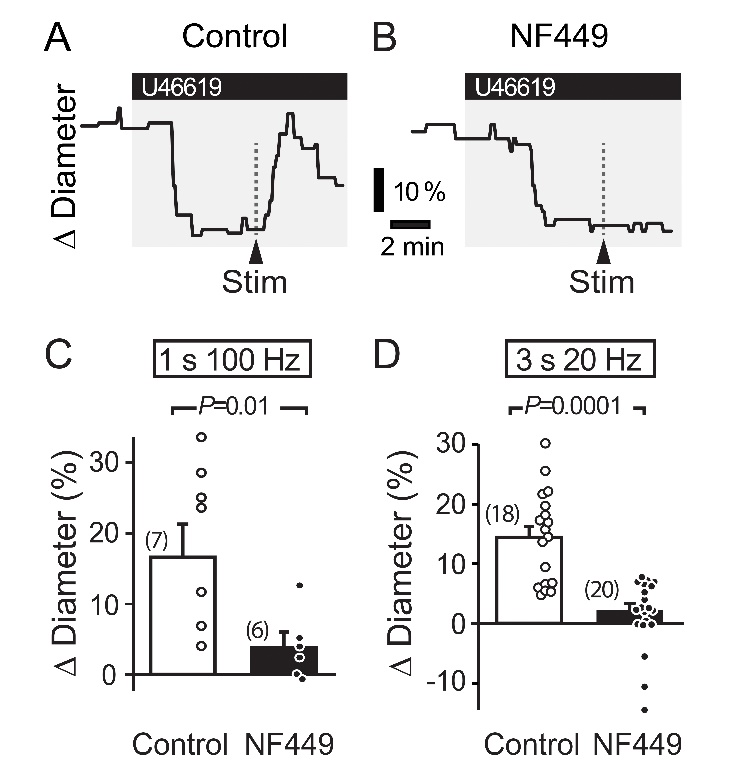


**Supplementary Figure 1.** **Stimulation-evoked capillary dilation in adult rat cortex depends on P2X1 receptors.** **A-B**. Capillary diameter changes in response to 200 nM U46619 and superimposed neuronal stimulation (Stim; 1s 100 Hz) in a control condition **(A)** and in the presence of 100 nM NF449, a potent P2X1 blocker **(B)**. **C-D**. Summary data demonstrating the effect of NF449 on capillary diameter evoked by a 1 s 100 Hz high-frequency stimulation (**C**) and a 3 s 20 Hz low-frequency stimulation **(D)**. *P* values obtained from Student’s t-tests. Number in parentheses above each bar indicates *N*.

**Supplementary Table 1.** Capillary diameter (µm; mean ± s.e.m.) at baseline, after pharmacological treatment, and following stimulation (Stim) for experiments plotted in **Figure 1** and **Figure 3**. No significant differences were detected in baseline diameter between groups (naïve vs. MCAO-Contra vs. MCAO-Ipsi) in any experiment.

|  | | **Baseline (**µm,) | **U46619** | **U+Drug** | **Stim** |
| --- | --- | --- | --- | --- | --- |
| **U46619 only** | Naïve | 5.6 ± 0.4 | 4.9 ± 0.4 | n/a | 5.6 ± 0.4 |
|  | MCAO Contra | 5.6 ± 0.4 | 5.1 ± 0.4 | n/a | 5.7 ± 0.5 |
|  | MCAO Ipsi | 5.4 ± 0.5 | 4.8 ± 0.6 | n/a | 5.0 ± 0.6 |
| **HET0016** | Naïve | 5.5 ± 0.3 | 5.0 ± 0.4 | 4.7 ± 0.4 | 5.2 ± 0.5 |
|  | MCAO Contra | 5.1 ± 0.5 | 4.6 ± 0.4 | 4.5 ± 0.5 | 5.0 ± 0.5 |
|  | MCAO Ipsi | 5.3 ± 0.4 | 4.9 ± 0.5 | 4.9 ± 0.5 | 5.4 ± 0.5 |
| **L-161,982** | Naïve | 5.7 ± 0.3 | 5.0 ± 0.2 | 4.9 ± 0.2 | 5.0 ± 0.2 |
|  | MCAO Contra | 4.9 ± 0.3 | 4.2 ± 0.3 | 4.1 ± 0.3 | 4.2 ± 0.3 |
|  | MCAO Ipsi | 5.3 ± 0.3 | 4.6 ± 0.3 | 4.4 ± 0.3 | 4.2 ± 0.3 |
| **L-161,982 + HET0016** | Naïve | 4.6 ± 0.5 | 4.0 ± 0.6 | 4.0 ± 0.6 | 4.1 ± 0.5 |
|  | MCAO Contra | 6.1 ± 0.4 | 5.4 ± 0.4 | 5.5 ± 0.4 | 5.6 ± 0.4 |
|  | MCAO Ipsi | 5.2 ± 0.6 | 4.4 ± 0.5 | 4.4 ± 0.6 | 4.5 ± 0.6 |
| **DETA-NONOate** | Naïve | 6.1 ± 0.3 | 5.4 ± 0.3 | 5.3 ± 0.3 | 5.9 ± 0.3 |
|  | MCAO Contra | 5.9 ± 0.4 | 5.1 ± 0.4 | 5.2 ± 0.4 | 5.7 ± 0.5 |
|  | MCAO Ipsi | 5.7 ± 0.5 | 5.1 ± 0.5 | 4.9 ± 0.5 | 5.4 ± 0.5 |
